# Induction of cerebellar cortical neurogenesis immediately following valproic acid exposure in ferret kits

**DOI:** 10.3389/fnins.2023.1318688

**Published:** 2023-12-07

**Authors:** Shiori Kamiya, Tetsuya Kobayashi, Kazuhiko Sawada

**Affiliations:** ^1^Department of Regulation Biology, Graduate School of Sciences and Engineering, Saitama University, Saitama, Japan; ^2^Department of Nutrition, Faculty of Medical and Health Sciences, Tsukuba International University, Tsuchiura, Japan

**Keywords:** cerebellum, valproic acid, neurogenesis, ferret, immunohistochemistry

## Abstract

**Introduction:**

Valproic acid (VPA) is an anticonvulsant/antiepileptic drug that regulates neurogenesis. Its effects vary depending on the timing of exposure and the types of neural progenitors involved. Neonatal exposure to VPA causes autism spectrum disorder-like behaviors in some mammalian species, including ferrets. Ferrets experience the cerebellar cortical histogenesis during early postnatal period. However, no studies have evaluated the effect of VPA on cerebellar corticohistogenesis. The present study aimed to determine the effects of VPA exposure on the developing cerebellar cortex in ferret kits with a particular focus on the cortical neurogenesis.

**Methods:**

The experimental kits each received an intraperitoneal injection of VPA, 200 μg/g body weight, on postnatal days 6 and 7. EdU and BrdU were administered on postnatal days 5 and 7, respectively, to label cells proliferating prior to and following exposure to VPA.

**Results:**

We found that 2 h post BrdU injection, BrdU-labeled cells were abundantly distributed in the internal granular layer (IGL), whereas EdU-labeled cells were primarily relegated to the inner pre-migratory zone of the external granular layer (EGL). The density of BrdU-single-labeled cells was significantly lower in the EGL and significantly higher in the IGL of the VPA-exposed group, as compared to the control group. Immunostaining for doublecortin, a marker of immature neurons, was observed in BrdU-single-labeled cells in the IGL of the VPA-exposed group, which was significantly higher than that observed in the control group. EdU-single-labeled cells that had proliferated prior to VPA exposure were also detected in the IGL. While the cell density remained unchanged, significant changes were observed in the proportions of EdU-single-labeled cells immunostained with marker antigens; higher proportion of PCNA immunostaining, but lower proportion of S100 immunostaining in the VPA-exposed group compared to the control group.

**Discussion:**

These findings suggest the presence of progenitors in the IGL of the developing cerebellar cortex in ferret kits. We called them “internal granular progenitors.” The progenitors may proliferate in response to VPA, leading the differentiated lineage more toward neurons than to glial cells. Thus, VPA may facilitate the differentiative division of internal granular progenitors to produce cerebellar granular neurons.

## Introduction

The cerebellum, which plays a role in several functions, including the regulation muscle tone, the integration of sensory and motor functions, and cognition (Hu et al., 2008), is composed of three cortical layers, the molecular layer (ML), the Purkinje cell layer (PCL), and the internal granular layer (IGL). The external granular layer (EGL), which contains external granular precursors (EGPs) that provide a source of granular neurons in the IGL, transiently emerges during cerebellar corticohistogenesis in rodents (Fujita et al., 1966; Altman, 1969, 1972; Iulianella et al., 2019; Consalez et al., 2021). Although ferrets experience a trajectory of cerebellar corticohistogenesis similar to that of rodents (Christensson et al., 2007; Kamiya and Sawada, 2021), relatively slower development and maturation of the cerebellar cortex with differential expression patterns of neurogenesis staging markers are observed in ferrets when compared to mice (Kamiya and Sawada, 2021; Sawada and Kamiya, 2023). Paired box 6 (Pax6), for example, is expressed primarily in differentiating/migrating granular neurons in ferrets, but in both the EGPs and differentiating/migrating granular neurons in mice (Sawada and Kamiya, 2023). These findings indicate that ferrets have characteristics advantageous for the investigation of the details of cerebellar cortical histogenesis, which are not seen in rodents.

Valproic acid (VPA), an anticonvulsant/antiepileptic drug, inhibits the activity of histone deacetylases 1 and 2 (Phiel et al., 2001). Exposure to VPA during the fetal or neonatal period can result in autism spectrum disorder (ASD)-like behaviors, such as deficits in social interactions, in various mammalian species, including rats (Mychasiuk et al., 2012; Favre et al., 2013), mice (Yochum et al., 2008; Burenkova et al., 2014), marmosets (Yasue et al., 2018), and ferrets (Krahe et al., 2016). These ASD-like behaviors are involved in abnormalities in the central nervous system (CNS), such as cerebral cortical thickening (Wood et al., 2014; Fujimura et al., 2016; Sawada et al., 2021b) or thinning (Hara et al., 2012), an increased number of cerebral cortical neurons (Sabers et al., 2014), sulcogyrogenesis abnormalities (Harden et al., 2004; Jou et al., 2010; Wallace et al., 2013; Yang et al., 2016; Sawada et al., 2021b), and an increased density of dentate gyrus progenitors in the hippocampus (Sawada et al., 2021a). VPA, for example, affects the adult hippocampus by promoting the proliferation of the dentate gyrus progenitors through the activation of the Wnt/β-catenin pathway in Alzheimer’s disease model mice (Zeng et al., 2019), or facilitates neural differentiation by inhibiting histone deacetylases 1 and 2 activity in rats (Hsieh et al., 2004). VPA also promotes the proliferation of intermediate progenitors and/or basal radial glia, which appear transiently in the subventricular zone of the developing cerebral cortex (Fujimura et al., 2016; Sawada, 2022).

Although VPA influences the developing cerebellum to induce cerebellar hypoplasia (Main and Kulesza, 2017; Shona et al., 2018), maldevelopment of Purkinje cells (Main and Kulesza, 2017), and apoptosis of the EGPs (Yochum et al., 2008), there have been no studies evaluating the effects of VPA on cerebellar cortical neurogenesis. The present study, therefore, aimed to determine the effects of VPA exposure on cerebellar corticohistogenesis based on changes to cerebellar cortical neurogenesis. We used ferret kits as a model animal to use immunohistochemical procedures to easily distinguish the different stages of granular neurogenesis in the developing cerebellar cortex (Sawada and Kamiya, 2023).

## Materials and methods

### Animals

We purchased six pregnant ferrets from Japan SLC Co. (Hamamatsu, Japan), from which eight naturally delivered male offspring were utilized for the present study. The kits were reared with lactating mothers (4–6 kits/mother) in stainless steel cages (80 cm × 50 cm × 35 cm) maintained at 21.5 ± 2.5°C with 12 h artificial illumination in the Facility of Animal Breeding, Nakaimizu Laboratory, Japan SLC. All lactating mothers were fed a pellet diet (high-density ferret diet 5 L14; PMI Feeds, Inc. St. Louis, MO, United States), and tap water *ad libitum*. All ferret kits received 5-ethynyl-2′-deoxyuridine (EdU; Sigma-Aldrich, St. Louis, MO, United States) intraperitoneally at 30 μg/g body weight on postnatal day (PD) 5, and 5-bromo-2′-deoxyuridine (BrdU; Sigma-Aldrich) intraperitoneally at 30 μg/g body weight on PD 7, while four kits received an intraperitoneal injection of VPA (Sigma-Aldrich) at 200 μg/g body weight on PDs 6 and 7, corresponding to the start of EGL expansion in the developing ferret cerebellar cortex (Kamiya and Sawada, 2021). The second VPA injection was administered simultaneously with the BrdU. The four kits that did not receive VPA were used as controls.

At 2 h post-BrdU injection, all kits received an intracardiac perfusion with 4% paraformaldehyde (Merck, Darmstadt, Germany) in phosphate buffered saline (PBS) (pH 7.4), under inhalation anesthesia with ~2% isoflurane (Fujifilm Wako Pure Chemicals, Osaka, Japan). The kit brains were removed from the skulls and immersed in the same fixative prior to study.

### Preparation of tissue sections

The cerebella were immersed in 30% sucrose-PBS solution overnight and then embedded in an optimal cutting temperature compound (Sakura Finetech Japan, Tokyo, Japan) at −70°C. After embedding and freezing, 100 μm thick sagittal sections taken from around the midsagittal plane were made using a Retratome (REM-700; Yamato Koki, Asaka, Japan) with a refrigeration unit (MC-802A; Yamato Koki).

### Immunofluorescence staining procedures

All immunofluorescence staining procedures were performed using the floating section method. All sections were heated in Antigen Retrieval Reagent Universal (R&D Systems, Minneapolis, MN, United States) at 90°C for 30 min. After cooling, the sections were incubated with 0.1% TritonX-100-PBS solution at 37°C for 1 h, followed by EdU detection using the Click-iT EdU Alexa Fluor Imaging Kit (Thermo Fisher Scientific, Waltham, MA, United States) at 37°C for 2 h. After washing with PBS, the sections were exposed to primary antibodies diluted in PBS containing 10% normal horse serum (Funakoshi, Tokyo, Japan) containing 0.1% TritonX-100, and then they were incubated at 4°C overnight. The primary antibodies used, which have been found to react specifically with ferret brain tissue (Kamiya and Sawada, 2021; Sawada and Kamiya, 2023), are listed in Supplementary Table S1. The sections were then incubated with the appropriate combination of secondary antibodies, listed in Supplementary Table S2, at 37°C for 2 h. When biotinylated anti-mouse IgG was used as a secondary antibody, the sections were treated with Alexa555-conjugated streptavidin (1:500, S21381; Thermo Fisher Scientific, Waltham, MA, United States) at 37°C for 2 h.

### Evaluation of cell density

Serial sectioned digital images were captured with a 20× object using an Axio Imager M2 ApoTome.2 (Zeiss, Gottingen, Germany) equipped with an AxioCam MRm camera (Zeiss, Gottingen, Germany) operating on Zen 2.3 blue edition software (Zeiss). A total of 10 serial sectioned images were acquired at a depth of 1 μm from the most superficial plane, where EdU and BrdU labeling with immunostaining for various markers were obtained. A set of sectional images- 4 μm apart in the Z-direction (the 3rd and 7th from the superficial slices of the acquired images)- were selected as the lookup and reference images, respectively. The densities of thymidine analog-labeled and immunostained cells were estimated with the dissector method, using systematic random sampling according to the procedure previously described by Sawada (2019).

In sections immunostained for each marker, frames with 6 square boxes (box size = 40 × 40 μm) were used to systematically select regions of interest (ROIs) randomly superimposed on the EGL, ML/PCL, and IGL of lobule V of the vermis at the deep portion of the primary fissure in both the lookup and reference images. Thymidine analog-labeled or immunostained cells were counted within the ROIs using the “forbidden line” rule (Gundersen, 1977). The densities were estimated using the following formula: [cell density = Qn−/(a × b × t)] [Qn- = total number of thymidine analog-labeled and/or immunostained cells observed in the lookup, but not the reference images; a = 6, total number of ROIs in the lookup images per animal; b = 40 × 40 μm, area of the counting box; and t = distance between the lookup and reference images (4 μm)]. The proportions of immunostained EdU-single-, BrdU-single-, and EdU/BrdU-double-labeled cells were calculated by totaling the number of cells counted within all ROIs from all of the animals in each group.

### Statistical analysis

The cell densities of thymidine analog-labeled and immunostained cells were statistically evaluated using repeated-measures two-way analysis of variance (ANOVA) and simple main effects, with the cerebellar cortical layers (EGL, ML/PCL, IGL) and groups (VPA-exposed and control groups) designated as the factors evaluated. For *post-hoc* testing, Scheffe’s test was used to detect differences between the groups and/or interactions between the group and cerebellar cortical layers, with *p* < 0.05 set as statistically significant. The proportion of immunolabeled cells in thymidine analog-labeled cells was statistically evaluated using the χ^2^ test, for which the total number of EdU-single-, BrdU-single-, and EdU/BrdU-double-labeled cells was defined as “n.”

## Results

### Distribution and density of EdU- and/or BrdU-labeled cells

**Figure 1:**
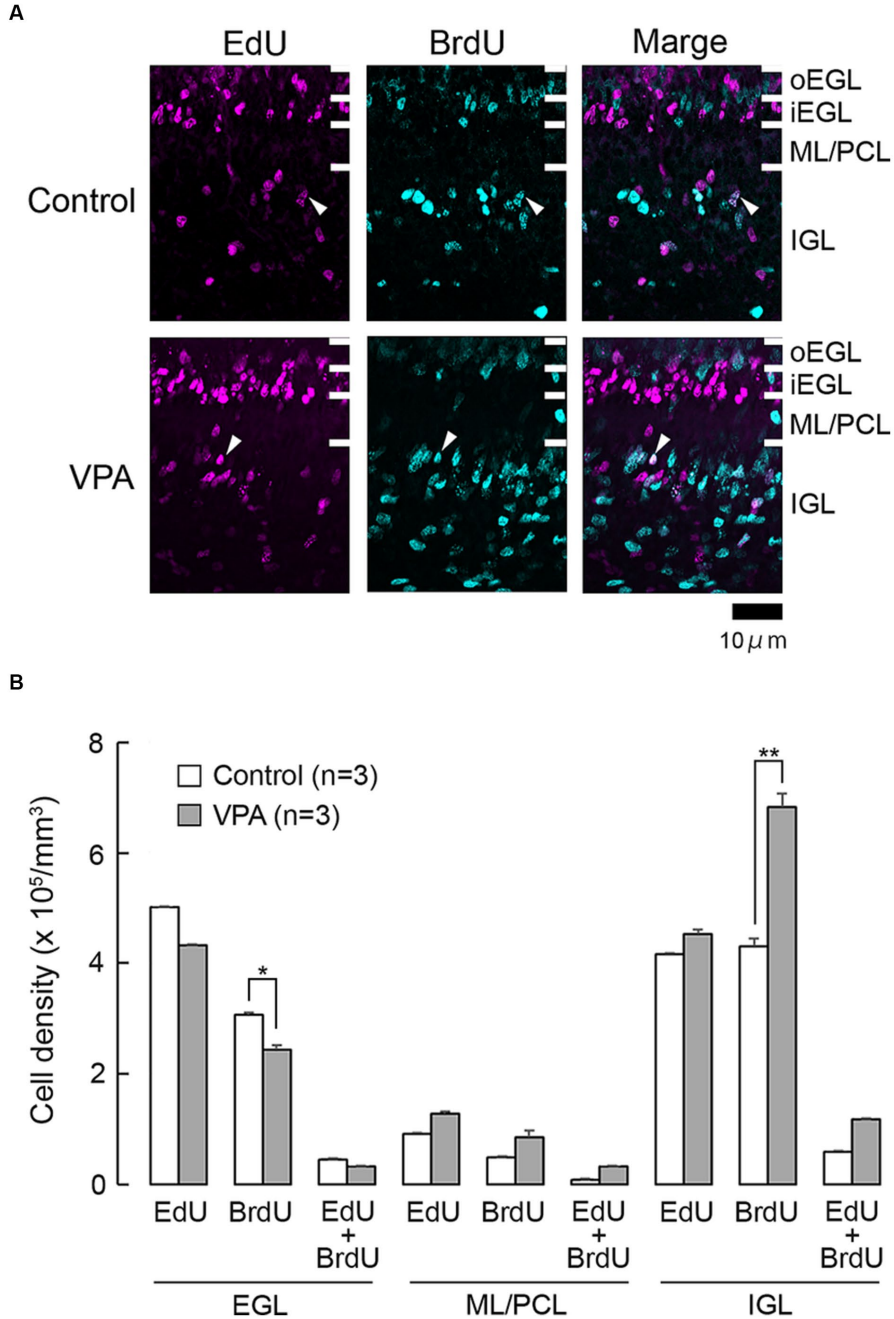
EdU and BrdU labeling in the cerebellar cortex of VPA-exposed and control ferrets on postnatal day 7. **(A)** EdU and BrdU labeling in the cerebellar cortex. Closed arrowheads, EdU/BrdU-double-labeled cells. **(B)** Bar graph of densities of EdU-single-, BrdU-single-, and EdU/BrdU-double-labeled cells in cerebellar cortical layers. Data are shown as mean ± standard error; significance is indicated using Scheffe’s test at *p < 0.05 and **p < 0.01. EGL, external granular layer; iEGL, inner external granular layer; IGL, internal granular layer; ML, molecular layer; oEGL, outer external granular layer; PCL, Purkinje cell layer.

**Figure 2:**
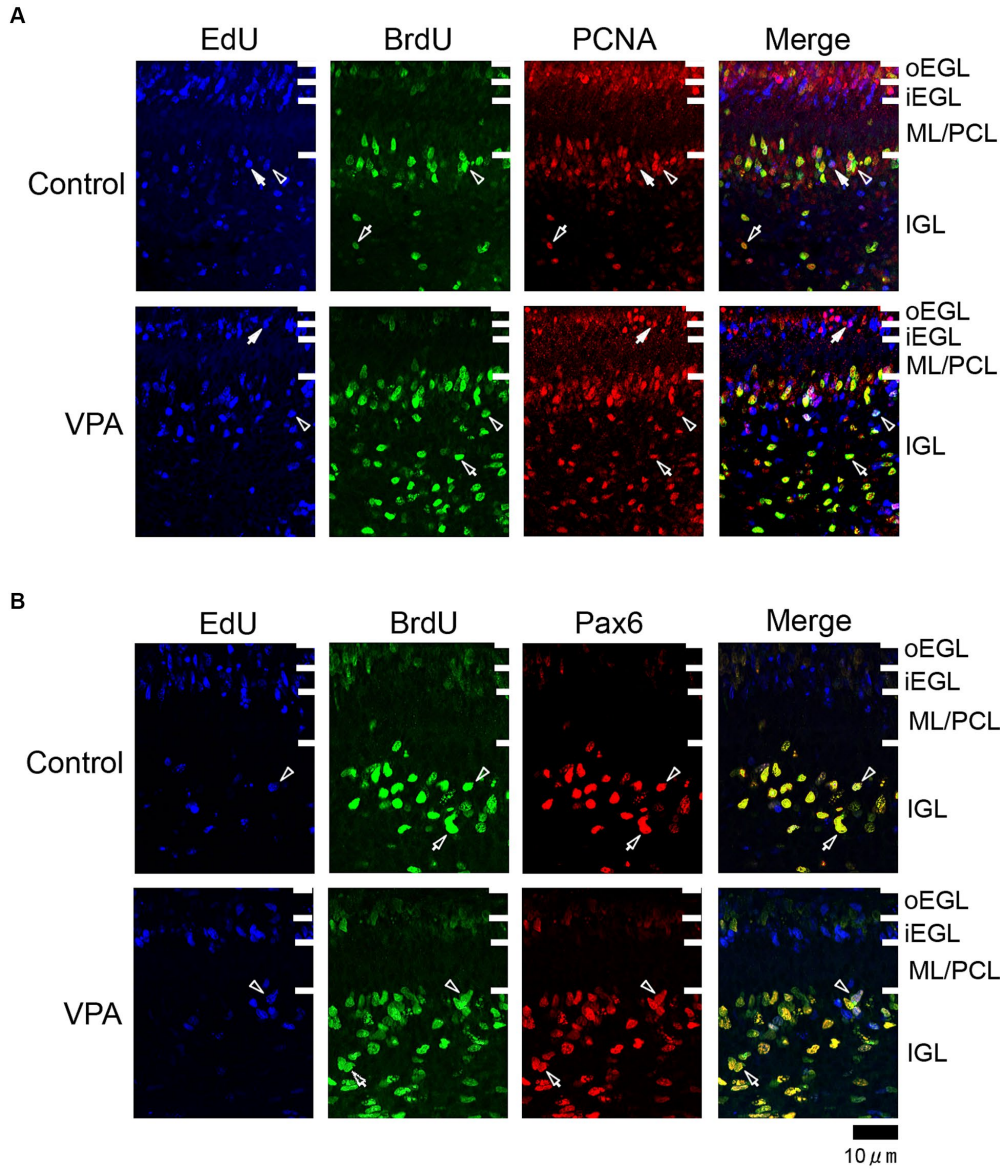
Immunofluorescence for PCNA and Pax6 with EdU and BrdU labeling in the cerebellar cortex of VPA-exposed and control ferrets on postnatal day 7. **(A)** PCNA immunofluorescence staining with EdU and BrdU labeling in the cerebellar cortex. Open arrowheads indicate EdU/BrdU-double-labeled PCNA-immunopositive cells; open arrows indicate BrdU-single-labeled PCNA-immunopositive cells; closed arrows indicate EdU-single-labeled PCNA-immunopositive cells. **(B)** Pax6 immunofluorescence staining with EdU and BrdU labeling. Open arrowheads indicate EdU/BrdU-double-labeled Pax6-immunopositive cells; open arrows indicate BrdU-single-labeled Pax6-immunopositive cells. iEGL, inner external granular layer; IGL, internal granular layer; ML, molecular layer; PCL, Purkinje cell layer; oEGL, outer external granular layer.

Cells undergoing S phase 24 h prior to the first administration of VPA on PD 6 were labeled with EdU. While the majority of these cells were single-labeled with EdU, some were double-labeled with BrdU (Figure 1A). EdU-single-labeled cells were observed in all layers of the premature cerebellar cortex, including the EGL, ML/PCL, and IGL (Figure 1A). EdU-single-labeled cells in the EGL were tightly arranged, particularly in the inner zone (iEGL), indicating EdU labeling in pre-migratory EGPs. EdU-single-labeled cells were sparse in the ML/PCL while they were abundant in the IGL (Figure 1A). These cells were immunoreactive to proliferating cell nuclear antigen (PCNA) (Figure 2A). We called PCNA-expressing cells in the IGL “internal granular progenitors (IGPs),” because nestin expression was detected some of them on PD 7 (Sawada and Kamiya, 2023). EdU/BrdU-double-labeled cells, which underwent the S phase again 48 h after the injection of EdU, were sparse in the EGL, ML/PCL, and IGL (Figure 1A). These may be self-renewing EGPs or IGPs on PD 7. BrdU-single-labeled cells were primarily observed in the outer proliferating zone of the EGL (oEGL) and IGL, while they were sparse in the ML/PCL (Figure 1A). Because post-mitotic EGPs are retained for 20–48 h in the iEGL before radial migration (Komuro et al., 2001), BrdU labeling in the IGL may appear in proliferating IGPs on PD 7.

The densities of EdU-single-, BrdU-single-, and EdU/BrdU-double-labeled cells in each layer of the cerebellar cortex were estimated according to the procedure previously described by of Sawada (2019), and the results are shown in Figure 1B. The densities of EdU-single- and EdU/BrdU-double-labeled cells did not differ significantly between the two groups in any of the cerebellar cortical layers (Figure 1B). In contrast, repeated-measures two-way ANOVA revealed significant differences in the density of BrdU-single-labeled cells between the VPA-exposed and control groups [*F*_(1, 4)_ = 14.669; *p* < 0.05], as well as an interaction between the cerebellar cortical layers and groups [*F*_(1, 4)_ = 17.664; *p* < 0.001]. Scheffe’s test indicated that the density of BrdU-single-labeled cells in the VPA-exposed group was significantly lower in the EGL (*p* < 0.05), while it was significantly higher in the IGL (*p* < 0.001), than in the control group (Figure 1B). The effects of VPA on cell proliferation, therefore, varied based on the type of cerebellar cortical precursors or progenitors.

### Proportion of immunostaining for neurogenesis markers in thymidine analog-labeled cells

EdU and BrdU labeling was performed using immunofluorescence staining for numerous neurogenesis marker antigens, such as PCNA, Pax6, doublecortin (DCX), and S100. Since the present study independently estimated the proportion of each marker immunostained in thymidine analog-labeled cells, it was unclear whether the expression of each marker antigen overlapped or not.

PCNA immunostaining was observed in the EGPs of the oEGL in both groups (Figure 2A). EdU-single-labeled EGPs were observed primarily in the iEGL. The proportion of PCNA immunostaining was low in both groups, but the difference was not detected (Table 1). EdU-single-labeled EGPs, therefore, which proliferated on PD 5, were largely in a pre-migratory state with attenuated PCNA expression on PD 7. PCNA immunostaining also appeared in BrdU-labeled IGP located in both the PCL and the IGL adjacent to the PCL (Figure 2A). PCNA immunostaining in the BrdU-single-labeled IGPs was >50% in the ML/PCL and >80% in the IGL, without significant differences between the VPA-exposed and control groups (Tables 2, 3). In contrast, the proportion of PCNA immunostaining in EdU-single-labeled IGPs was 40.3% in the VPA-exposed group, significantly higher than that in the control group (18.2%; Table 3). The proportion of PCNA immunostaining in EdU/BrdU-double-labeled IGPs was also significantly greater in the VPA-exposed than the control group (Table 3). Thus, PCNA expression was sustained in post-proliferative EdU-labeled IGPs.

**Table 1:** Percentages of immunostained cells for various markers in EdU-single-, BrdU-single- and EdU/BrdU-double-labeled cells in external granular layer of cerebellar cortex.

	EGL			
	Control		VPA	
**EdU-single-labeled cells**				
% of PCNA	12.1%	(16/132)	9.4%	(8/85)
% of Pax6	10.3%	(11/107)	5.1%	(6/118)
% of S100	0%	(0/9)	0%	(0/18)
% of DCX	2.5%	(2/81)	6.3%	(4/63)
**BrdU-single-labeled cells**				
% of PCNA	43.8%	(32/73)	25.9%	(7/27)
% of Pax6	100%	(105/105)	78.0%	(64/82)**
% of S100	0%	(0/71)	0%	(0/85)
% of DCX	4.9%	(3/61)	5.3%	(2/38)
**EdU/BrdU-double-labeled cells**				
% of PCNA	100%	(2/2)	66.7%	(2/3)
% of Pax6	64.3%	(9/14)	50.0%	(5/10)
% of S100	0%	(0/9)	0%	(0/18)
% of DCX	20.0%	(1/5)	ND	

Percentages are calculated by summing each labeled cell counted within all ROIs from the external granular layer (EGL) of the cerebellar cortex from three ferrets. The number of each labeled cell for calculating the percentages is shown in parentheses. **p < 0.01 vs. control group (χ^2^ test). ND, not detected.

**Table 2:** Percentages of immunostained cells for various markers in EdU-single, BrdU-single, and EdU/BrdU-double-labeled cells in molecular and Purkinje cell layers of cerebellar cortex.

	ML/PCL			
	Control		VPA	
**EdU-single-labeled cells**				
% of PCNA	25.8%	(8/31)	9.1%	(3/33)
% of Pax6	0%	(0/18)	2.6%	(1/38)
% of S100	0%	(0/11)	16.7%	(2/12)
% of DCX	12.5%	(1/8)	0%	(0/26)
**BrdU-single-labeled cells**				
% of PCNA	50.0%	(6/12)	57.6%	(19/33)
% of Pax6	71.4%	(5/7)	60.0%	(21/35)
% of S100	0%	(0/2)	42.9%	(3/7)
% of DCX	9.1%	(1/11)	0%	(0/14)
**EdU/BrdU-double-labeled cells**				
% of PCNA	0%	(0/1)	0%	(0/1)
% of Pax6	ND		100%	(1/1)
% of S100	ND		0%	(0/1)
% of DCX	50.0%	(1/2)	0%	(0/3)

Percentages are calculated by summing each labeled cell counted within all ROIs from molecular layer (ML) and Purkinje cell layer (PCL) of cerebellar cortex from three ferrets. The number of each labeled cell for calculating the percentages is shown in parentheses. ND, not detected.

**Table 3:** Percentages of immunostained cells for various markers in EdU-single, BrdU-single, and EdU/BrdU-double-labeled cells in internal granular layer of cerebellar cortex.

	IGL			
	Control		VPA	
**EdU-single-labeled cells**				
% of PCNA	18.2%	(22/121)	40.3%	(50/124)**
% of Pax6	19.0%	(15/79)	15.0%	(15/100)
% of S100	49.6%	(57/115)	37.3%	(44/118)**
% of DCX	11.3%	(9/80)	8.5%	(5/59)
**BrdU-single-labeled cells**				
% of PCNA	80.5%	(107/133)	83.4%	(136/163)
% of Pax6	91.2%	(83/91)	95.2%	(160/168)
% of S100	52.9%	(54/102)	45.2%	(66/146)
% of DCX	6.8%	(5/74)	28.2%	(31/110)**
**EdU/BrdU-double-labeled cells**				
% of PCNA	40.0%	(8/20)	79.4%	(27/34)*
% of Pax6	75.0%	(9/12)	75.0%	(15/20)
% of S100	51.6%	(16/31)	56.5%	(26/46)
% of DCX	0%	(0/21)	8.3%	(1/12)

Percentages are calculated by summing each labeled cell counted within all ROIs from inner granular layer (IGL). of cerebellar cortex from three ferrets. The number of each labeled cell for calculating the percentages is shown in parentheses. *p < 0.05, **p < 0.01. vs. control group (χ^2^ test).

In the present study, Pax6 immunostaining overlapped with BrdU labeling in the majority of EGPs or IGPs in the cerebellar cortex of ferrets on PD 7 (Figure 2B). Many BrdU+ progenitors exhibited Pax6 immunoreactivity also in other brain regions, for example, the subventricular zone of the cerebral cortex, with the sections prepared from the same brain sample of control ferrets used in this study (Supplementary Figure S1). In these sections, some Pax6+ progenitors were not labeled with BrdU (Supplementary Figure S1), indicating that the overlap of these two in the ferret brain tissue was not an artifact. Weak Pax6 immunostaining was observed in some EGPs aligned with the oEGL (Figure 2B). Since EdU-single-labeled EGPs, which proliferated on PD 5, were aligned tightly through the iEGL but were sparse in the oEGL (Figure 2B), the proportion of Pax6 immunostaining was <10% of EdU-single-labeled EGPs in both groups of ferrets (Table 1). In contrast, BrdU-single-labeled EGPs, which proliferated on PD 7, were primarily localized in the oEGL (Figure 2B). All BrdU-single-labeled EGPs were Pax6 immunopositive in the control group. A significantly lower proportion of Pax6 immunostaining (78.0%) was observed in the VPA-exposed group (Table 1). Pax6 expression, therefore, appeared weak in proliferating EGPs in the oEGL, and subsequently disappeared in pre-migratory EGPs aligned in the iEGL. However, in the IGL, Pax6 immunostaining was observed in >90% of BrdU-single-labeled cells, and in >75% of EdU/BrdU-double-labeled cells in both groups of ferrets. These differences were not significant (Table 3). Since post-mitotic EGPs are retained for 20–48 h in the iEGL before radial migration (Komuro et al., 2001), Pax6 expression in the IGL may be obtained in IGPs. A large population of IGPs proliferated on PD 7 (BrdU-labeled) was Pax6 immunopositive, suggesting that post-proliferative IGPs begin to differentiate into granular neurons. Thus, IGPs may be also a source of granular neurons distinct from the EGPs in ferrets.

**Figure 3:**
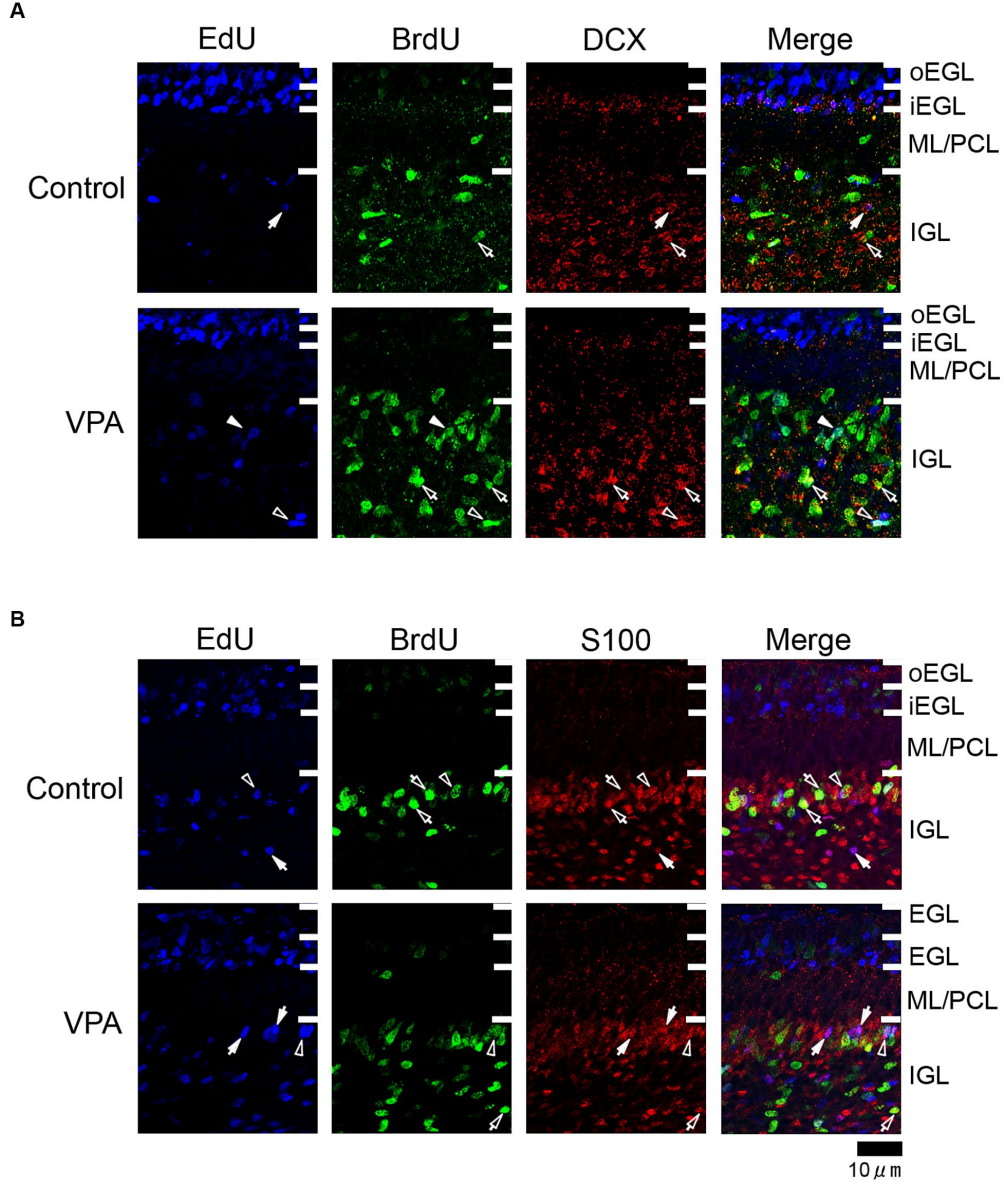
Immunofluorescence for DCX and S100 in the cerebellar cortex of VPA-exposed and control ferrets on postnatal day 7. **(A)** DCX immunofluorescence staining with EdU and BrdU labeling in the cerebellar cortex. Open arrowheads indicate EdU/BrdU-double-labeled DCX-immunopositive cells; open arrows indicate BrdU-single-labeled DCX-immunopositive cells; closed arrows indicate EdU-single-labeled DCX-immunopositive cells. **(B)** S100 immunofluorescence staining with EdU and BrdU labeling. Open arrowheads indicate EdU/BrdU-double-labeled S100-immunopositive cells; open arrows indicate BrdU-single-labeled S100-immunopositive cells; closed arrows indicate EdU-single-labeled S100-immunopositive cells. iEGL, inner external granular layer; IGL, internal granular layer; ML, molecular layer; PCL, Purkinje cell layer; oEGL, outer external granular layer.

Immunostaining for DCX, a marker of immature neurons, was found mainly in the IGL of the VPA-exposed group (Figure 3A; Table 3). The proportion of DCX immunostaining was low in EdU-single-, BrdU-single-, and EdU/BrdU-double-labeled cells in both groups of ferrets. A significantly greater proportion of DCX immunostaining was observed in BrdU-single-labeled cells in the IGL of the VPA-exposed group (28.2%) than the controls (6.8%; Table 3). The enhanced differentiation of IGPs into granular neurons, therefore, was revealed by DCX and Pax6 immunostaining.

S100 immunopositive cells were abundant in the IGL adjacent to the PCL (Figure 3B), at approximately 37–50% of EdU-single-, 45–53% of BrdU-single-, and 51–56% of EdU/BrdU-double-labeled cells (Table 3). Approximately half of the IGPs generated on PDs 5 and 7, therefore, may have potent effects on glial cell lineage differentiation. The significantly lower proportion of S100 immunostaining in EdU-single-labeled cells in the VPA-exposed group (37.3%) than the control group (49.6%) in the IGL (Table 3). IGPs may differentiate into glial cells, which decreased under the influence of VPA.

### Density of cells immunopositive for neurogenesis markers

**Figure 4:**
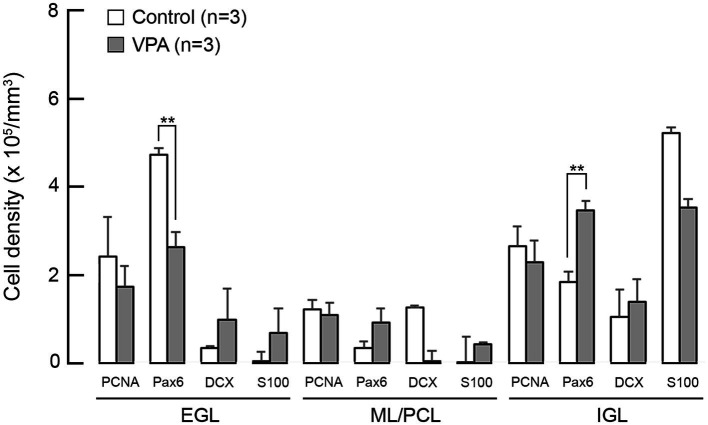
Bar graphs of density of cells immunostained for various neurogenesis markers in the cerebellar cortex of VPA-exposed and control ferrets on postnatal day 7. Data are shown as mean ± standard error. Significance is indicated using Scheffe’s test at *p < 0.05 and **p < 0.01. EGL, external granular layer; IGL, internal granular layer; ML, molecular layer; PCL, Purkinje cell layer.

The densities of cells immunostained for neurogenesis staging markers in the cerebellar cortex on PD 7 (2 h after the second VPA injection in the VPA-exposed group) are shown in Figure 4. A repeated-measures two-way ANOVA demonstrated that there was a significant interaction between the cerebellar cortical layers and the groups [*F*_(1, 4)_ = 35.335; *p* < 0.001] on the Pax6 immunopositive cell density. Scheffe’s test showed that the density of Pax6 immunopositive cells was significantly lower in the EGL (*p* < 0.001), but significantly higher (*p* < 0.001) in the IGL, in the VPA-exposed group than in the control group (Figure 4). The densities of cells immunopositive for other markers, such as PCNA, DCX, and S100, were not significantly different between the VPA-exposed and control groups in any layers of the cerebellar cortex (Figure 4).

## Discussion

The cerebellum develops slower than other CNS regions, forming a cortical histoarchitecture during the early postnatal life in mice and ferrets (Altman, 1972; Christensson et al., 2007; Kamiya and Sawada, 2021). Although VPA regulates neurogenesis by modulating the proliferation, maintenance, and differentiation of neural progenitors, its effects vary, depending on the timing of the exposure and the types of neural progenitors (Hsieh et al., 2004; Wang et al., 2010; Fujimura et al., 2016; Wang et al., 2016; Sawada et al., 2021a; Sawada, 2022). The present study examined the effects of VPA exposure on cerebellar cortical neurogenesis in ferret kits, assessed by the expression patterns of neurogenesis staging markers. The administration of VPA inhibited the proliferation of EGPs, with preventing their transformation to the pre-migratory status. In contrast, IGPs, another source of granular neurons in ferrets distinct from EGPs, exhibited different responses to VPA from EGPs. While IGPs also had a potency to differentiate glial cells, IGPs may proliferate in response to VPA, leading the differentiation more toward granular neurons rather than to glial cells.

Cerebellar dysfunction caused by drugs, chemicals, injuries, and/or genetic insults results in a variety of neurological symptoms, such as ataxia, dystonia, and tremors, as well as neurodevelopmental and psychological disorders, such as ASD, schizophrenia, and attention deficit hyperactivity disorder (Lackey et al., 2018). It is well known that ASD-like symptoms are associated with cerebellar hypoplasia due to a reduced number of Purkinje cells (Kemper and Bauman, 1998), cerebellar neuroinflammation (Vargas et al., 2005), an altered number and morphology of deep cerebellar neurons (Kemper and Bauman, 1998), and reduced gray matter volume of the posterior cerebellum (Webb et al., 2009). The results of the present study indicated that neonatal VPA exposure suppressed the proliferation of EGPs while promoting the proliferation of IGPs toward the differentiation into granular neurons. While it remains unclear whether there is a difference in the contribution of granular neurons derived from the two distinct origins, namely EGPs and IGPs, to cerebellar function, the findings of the present study predict an overproduction of IGP-derived granular neurons induced by neonatal VPA exposure. An excitatory/inhibitory imbalance in neural networks, for example the overproduction of excitatory neurons in the upper layer of the cerebral cortex, is involved in ASD-like behavioral deficits (Rubenstein and Merzenich, 2003). Therefore, ASD-like social impairments, as observed in VPA-exposed neonatal ferrets (Krahe et al., 2016), may be the outcome of a disrupted excitatory/inhibitory balance in the cerebellar cortical network by enhanced granular neurogenesis from IGPs by VPA. However, we did not evaluate the number of granular neurons in the sexually matured ferrets of our VPA-exposed model. Further studies are required to clarify the overproduction of granular neurons in relation to ASD-like behavioral deficits in our VPA-exposed ferret model or in ferrets with a long-term exposure to VPA neonatally.

During postnatal cerebellar corticohistogenesis, the cerebellar interneurons such as Golgi, basket and stellate neurons were generated in the prospective white matter and migrated into their final locations (Galas et al., 2017). This study could not evaluate the effect of VPA on the generation of these cerebellar interneurons. This is a limitation in the current investigation that differentiating/migrating cerebellar interneurons were unidentifiable by immunohistochemical procedures. Therefore, we need to investigate the fate of IGPs, including the possibility that the cerebellar interneurons are differentiated.

## Conclusion

Granular neurons are the most abundant neuronal cell type in the cerebellum and generated from EGPs in many species of vertebrates (Iulianella et al., 2019; Consalez et al., 2021). The present findings revealed that novel progenitors, named “IGPs,” in the IGL of the developing cerebellar cortex of ferret kits. IGPs may be a secondary source of granular neurons distinct from EGPs and related to continuous expansion and sublobulation after the completion of the cerebellar cortical histogenesis in ferrets, unlike rodents (Kamiya and Sawada, 2021). Furthermore, the epigenetic regulation of neurogenesis from IGPs was revealed by VPA administration experiments. The present findings provide new insights into the development and evolution of the cerebellum in mammals. Investigating the existence of IGPs in primates including humans will aid in elucidating cerebellar functions such as memory and cognition.

## Data availability statement

The raw data supporting the conclusions of this article will be made available by the authors, without undue reservation.

## Ethics statement

The animal study was approved by Institutional Animal Care and Use Committee of Tsukuba International University. The study was conducted in accordance with the local legislation and institutional requirements.

## Author contributions

SK: Conceptualization, Formal analysis, Investigation, Methodology, Validation, Writing – original draft. TK: Supervision, Writing – review & editing. KS: Conceptualization, Funding acquisition, Project administration, Writing – review & editing.
