# Supplementary material for: Induction of cerebellar cortical neurogenesis immediately following valproic acid exposure in ferret kits
Source: Front Neurosci. 2023 Dec 7;17:1318688. doi: 10.3389/fnins.2023.1318688 (PMC10734798; doi:10.3389/fnins.2023.1318688)
Supplement: Supplementary file 1 [file Data_Sheet_1.PDF]

Table S1. Primary antibodies used in this study.

Antigens	Hosts	Concentration used	Cat#	RRDD	Source
BrdU	Rat monoclonal	1:500	ab6326	AB_305426	Abcam
BrdU	Sheep polyclonal	1:500	ab1893	AB_302659	Abcam
PCNA	Mouse monoclonal	1:500	MAB424	AB_95106	Millipore
Pax6	Mouse monoclonal	1:500	ab78545	AB_1566562	Abcam
DCX	Guinea pig polyclonal	1:500	AB2253	AB_1586992	Millipore
S100	Rabbit polyclonal	1:500	942001	AB_572261	ImmunoStar
