# Supplementary material for: Induction of cerebellar cortical neurogenesis immediately following valproic acid exposure in ferret kits
Source: Front Neurosci. 2023 Dec 7;17:1318688. doi: 10.3389/fnins.2023.1318688 (PMC10734798; doi:10.3389/fnins.2023.1318688)
Supplement: Supplementary file 2 [file Data_Sheet_2.PDF]

Table S2. Secondary antibodies used in this study.

Secondary antibody	Hosts	Concentration used	Cat#	Source
Alexa Fluor 555 anti-mouse	Donkey	1:500	A31570	Thermo Fisher Scientific
Alexa Fluor 647 anti-rabbit	Donkey	1:500	A31571	Thermo Fisher Scientific
Alexa Fluor 350 Anti-rat	Goat	1:500	A21093	Thermo Fisher Scientific
Alexa Fluor 350 Anti-sheep	Donkey	1:500	A21097	Abcam
