# Supplementary material for: Induction of cerebellar cortical neurogenesis immediately following valproic acid exposure in ferret kits
Source: Front Neurosci. 2023 Dec 7;17:1318688. doi: 10.3389/fnins.2023.1318688 (PMC10734798; doi:10.3389/fnins.2023.1318688)
Supplement: Supplementary file 3 [file Data_Sheet_3.PDF]

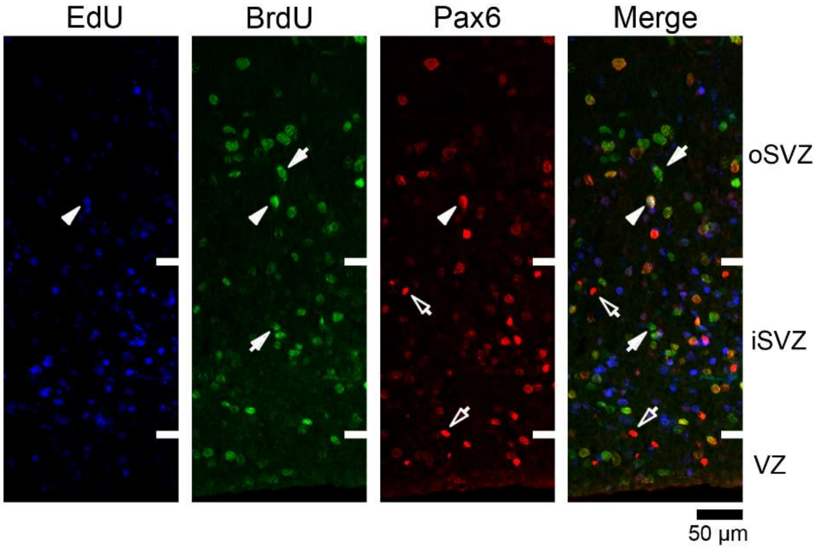

Figure S1. Immunofluorescence for Pax6 with EdU and BrdU labeling in the subventricular zone of the cerebral cortex of control ferrets on postnatal day 7. Immunofluorescence images were obtained from sections, which made from the same brain sample of control ferrets used in the present study. Closed arrowheads indicate EdU/BrdU-double-labeled Pax6-immunopositive progenitors; open arrows indicate Pax6-immunopositive progenitors; closed arrow indicate BrdU-single labeled progenitors. iSVZ, inner subventricular zone; oSVZ, outer subventricular zone; VZ, ventricular zone.
